# Altered Expression of Signaling Genes in Jurkat Cells upon FTY720 Induced Apoptosis

**DOI:** 10.3390/ijms11093087

**Published:** 2010-09-02

**Authors:** Fang Wang, Wenfeng Tan, Dunming Guo, Xiaomin Zhu, Keqing Qian, Shaoheng He

**Affiliations:** 1 The Lab of Cardiology, the First Affiliated Hospital of Nanjing Medical University, Nanjing 210029, China; E-Mail: fangwang7608@263.net; 2 Department of Rheumatology, the First Affiliated Hospital of Nanjing Medical University, Nanjing 210029, China; E-Mail: tanwenfeng2005@126.com; 3 Department of Orthopaedics, the First Affiliated Hospital of Nanjing Medical University, Nanjing 210029, China; E-Mail: jbjs3003@yahoo.com.cn; 4 The Clinical Experimental Center, the First Affiliated Hospital of Nanjing Medical University, Nanjing 210029, China; E-Mail: zhuxiaomin1226@126.com; 5 Department of Oncology, the Second Changzhou People’s Hospital, Affiliated to Nanjing Medical University, Changzhou 213003, China; E-Mail: cucu.2007@163.com

**Keywords:** FTY720, signal transduction, jurkat cell, apoptosis, cDNA microarray

## Abstract

FTY720, a novel immunosuppressant, has a marked activity in decreasing peripheral blood T lymphocytes upon oral administration. Recent investigations suggest that the action of FTY720 on lymphocytes may result from its ability to induce cell apoptosis. However, the cell signaling mechanism involved in the FTY720-induced cell apoptosis remains unclear. Here we examined the apoptotic signal pathways mediated by FTY720 in Jurkat cells using microarray analysis. The results showed that FTY720 can induce Jurkat cell apoptosis in a dose and time dependent manner as assessed by cell viability, Hoechst 33258 staining, Annexin V binding and DNA fragmentation tests. cDNA microarray analysis showed that 10 μM of FTY720 up-regulated 54 and down-regulated 10 genes in Jurkat cells among the 458 apoptotic genes examined following the 6 h incubation period. At least five-fold increased expression of modulator of apoptosis-1 (MOAP-1), vascular endothelial growth factor (VEGF), tumor necrosis factor receptor-associated factors (TRAF 6), Caspase 2 (CASP 2), E2F transcription factor 1 (E2F 1) and Casapse 5 (CASP 5) genes was observed in microarray analyses; these results were confirmed with reverse transcription polymerase chain reaction (RT-PCR) examination. Our findings suggest that the mitochondria related signaling pathways are the key pathways involved in the FTY720-induced apoptosis in Jurkat cells. And our results provide a new insight into the mechanism of FTY720, which allows us to draw the first simple diagram showing the potential pathways mediated by FTY720.

## 1. Introduction

The novel immunosuppressant FTY720, 2-amino-2-[2-(4-octylphenyl) ethyl]-1,3-propane-diol hydrochloride, is a synthetic compound that is chemically modified from ISP-1 ((2*S*,3*R*,4*R*)-(*E*)-2-amino-3,4-dihydroxy-2-hydroxymethyl-14-oxoeicos-6-enoic acid), a metabolite of the fungus *Isaria sinclair ii* [1]. It has been reported that FTY720 exhibits strong immunosuppressive activity in animal models of transplantation [2] and autoimmunity [3] without causing severe adverse reaction.

A striking feature of FTY720 is the induction of a marked decrease in the number of circulating lymphocytes and inhibition of lymphocyte influx into inflammatory sites [4]. Unlike conventional immunosuppressants, FTY720 does not impair the activation or proliferative functions of T cells and B cells [5]. It is believed that the powerful immunosuppressive activity of FTY720 is related to the significant decrease in the number of blood T lymphocytes upon oral administration. However, the mechanism for the decrease in lymphocytes induced by FTY720 is not well understood. At present, two reports attract most attention on the issue. Chiba *et al.* have demonstrated that the decrease in blood lymphocytes was mainly caused by FTY720-induced sequestration of lymphocytes into lymph nodes and Peyer’s patches [6]. However, Luo *et al.* reported that administration of FTY720 to *aly/aly* mice, which lack lymph nodes and Peyer’s patches, decreased the peripheral T lymphocytes in the same manner as in normal mice [7]. In addition, administration of FTY720 to C–C chemokine receptor 7 (CCR-7) knockout mice also induced a marked decrease of peripheral T lymphocytes [8]. These results indicate that some other mechanisms are likely to be involved in the immunosuppressive effect of FTY720. Several reports have showed that oral administration of FTY720 selectively induces apoptosis of mature peripheral blood T cells or cancer cells in animal models of organ transplantation and cancer, resulting in a marked decrease in the number of blood lymphocytes or cancer cells [2,9–11]. Furthermore, the ability of FTY720 to induce apoptosis in normal lymphocytes and cancer cells *in vitro* has been investigated [12–14]. FTY720-induced apoptosis appeared to be related to the activation of caspase cascade [15], bcl-associated signaling [16,17] independent of Fas [18], phospholipase C [14] and protein kinases [12]. In order to better understand the potential apoptotic pathways induced by FTY720, we analyzed apoptotic signaling genes in the present study.

It has been recognized that FTY720-induced cells apoptosis mostly focused on single gene expression. Because apoptosis is a complicated biological process involving a large number of genes, it is necessary to investigate FTY720-related apoptotic signaling genes as a whole in a defined experimental system. Microarray analysis has been used extensively for simultaneous analysis of transcript levels of thousands of genes in different physiological states of an organism, tissue, or cell over the last decade [19–21]. Such analyses have helped to identify numerous functional genes in many different cell types. We therefore employed the microarray technique in the present study to examine the signaling mechanism involved in FTY720-induced apoptosis in a human lymphoid T cell line, Jurkat cells.

## 2. Results and Discussion

### 2.1. FTY720 Reduces Cell Viability

**Figure 1:**
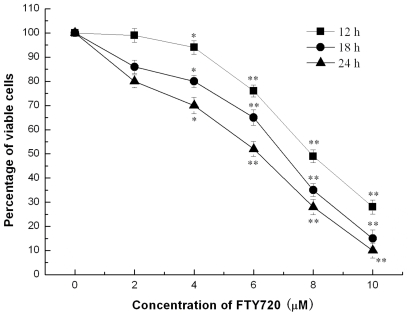
The effect of FTY720 on viability of Jurkat cells. Jurkat cells were incubated with various concentrations of FTY720 for 12, 18 and 24 h. MTT assay showed that FTY720 treatment caused a dramatic decrease in the viability of Jurkat cells in a dose– and time–dependent manner. The results are presented as the percentage reduction of cell viability in comparison to untreated cells. Data are expressed as mean ± S.D. of 3 independent experiments performed in duplicate. * *P* < 0.05, ** *P* < 0.01 *versus* the absorbance of untreated cells.

Jurkat cells were incubated with and without FTY720 at concentrations between 2 and 10 μM for 12, 18 and 24 h, respectively, and the cell viability was then assessed by Methyl thiazol tetrazolium (MTT) assay (Figure 1). It was observed that the cell viability was markedly reduced in a dose dependent manner starting from 4 μM of FTY720. At a dose of 10 μM, FTY720 eliminated cell viability by 88–90% after treatment for 24 h. FTY720 also showed a time-dependent reduction of Jurkat cell viability with the maximum reduction observed at 24 h after incubation (Figure 1).

### 2.2. FTY720 Induces Cell Apoptosis in Jurkat Cells

**Figure 2:**
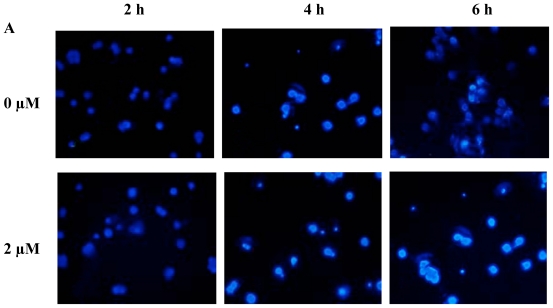

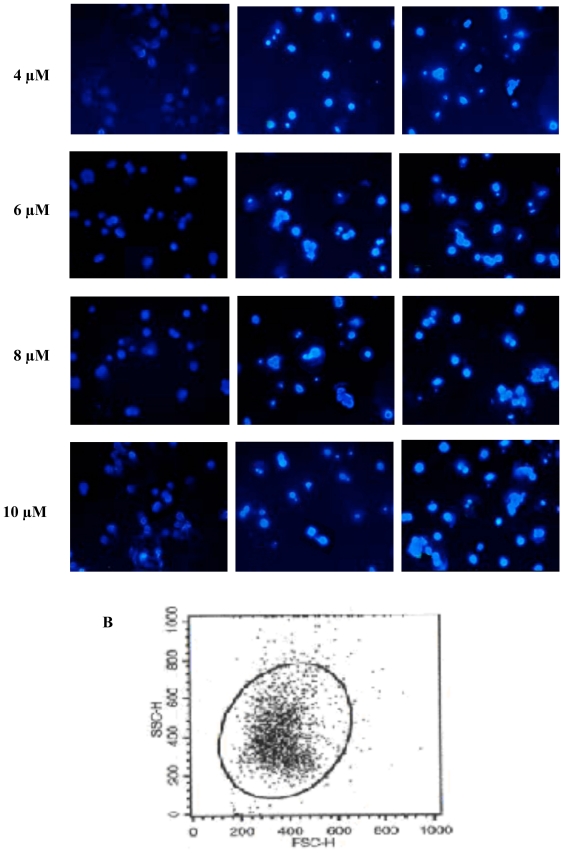

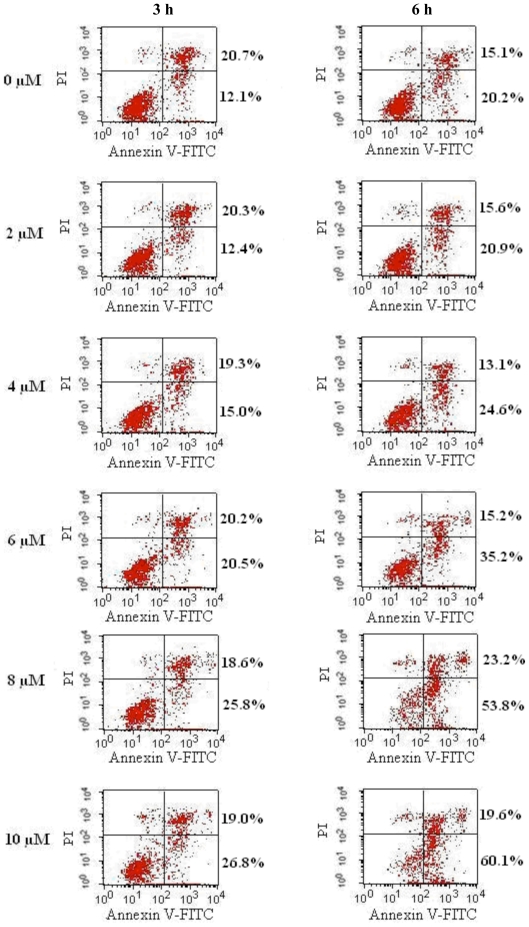

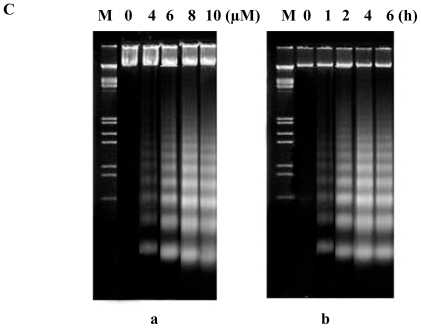
Detection of apoptosis of Jurkat cells induced by FTY720. (**A**) Jurkat cells were incubated with various concentrations of FTY720 for 2, 4 and 6 h before being stained with Hoechst 33258. Magnification = ×400. (**B**) Jurkat cells were incubated with various concentrations of FTY720 for 3 and 6 h before being stained with Annexin V/PI and analyzed by flow cytometry. (**C**) Agarose gel electrophoresis for DNA fragmentation (DNA ladder formation) in Jurkat cells induced by FTY720. a: Jurkat cells were incubated with various concentrations of FTY720 for 6 h; b: Jurkat cells were incubated with 10 μM FTY720 for 0, 1, 2, 4 and 6 h.

The typical apoptotic morphological changes such as condensed chromatin and shrunken, crimpled and condensed gray-blue nuclei were found in the FTY720-treated Jurkat cells after staining with Hoechst 33258. FTY720-induced apoptosis of Jurkat cells occurred at 2 h following incubation and became more apparent at 4 and 6 h. FTY720 induced apoptosis was dose-dependent, with the most apparent stain occurring after the addition of 10 μM FTY720 for 6 h (Figure 2A). Quantitative determination of FTY720 induced Jurkat cell apoptosis was performed by using Annexin V and **PI** staining. FTY720 induced a dose- and time-dependent apoptosis of Jurkat cells with up to 60.1% apoptotic cells observed following incubation with 10 μM FTY720 for 6 h (Figure 2B). A dose-dependent DNA fragmentation (DNA ladder formation) was observed by agarose gel electrophoresis following incubation of 0 to 10 μM FTY720 with Jurkat cells for 6 h. FTY720 induced DNA fragmentation was also time-dependent, with the most apparent bands being observed at 6 h following incubation with 10 μM FTY720 (Figure 2C).

### 2.3. Microarray Analysis

**Figure 3:**
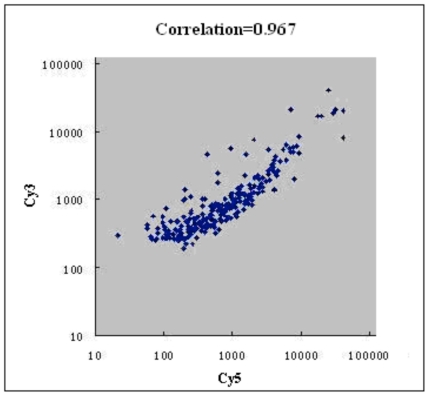
Scatter plot for the correlation of gene expression levels between FTY720 treated cells and untreated cells. The Cy3 (treated group) and Cy5 (untreated control) channel intensities from the two-color DNA microarray experiments were showed in the scatter plot. Variables appear a linear relationship, and the linear correlation between them is 0.967.

In order to obtain a comprehensive picture of the transcriptional changes caused by FTY720, total RNA from Jurkat cells (treated with 10 μM FTY720 for 6 h) was extracted for microarray analysis. The results showed that the expression of 64 genes (out of the 458 genes) was significantly altered by the presence of FTY720 in the Jurkat cells, among which 54 genes were up-regulated and 10 genes were down-regulated (Table 2). The scatter plot showed that the gene expression levels of FTY720 treated cells and untreated cells seemed to have a linear relationship, and the linear correlation between these two variables was 0.967 (Figure 3).

### 2.4. Validation of Microarray Data with RT-PCR and Western Blotting

**Figure 4:**
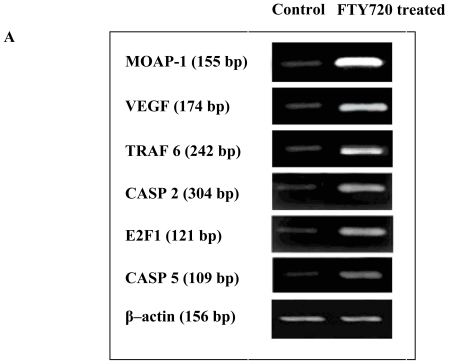

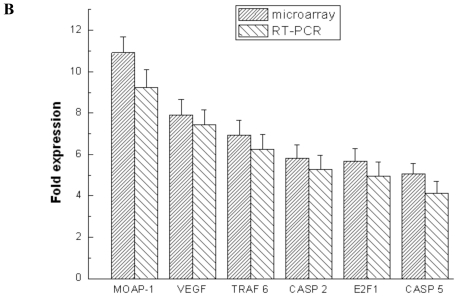
RT-PCR analyses of mRNA levels of six selected genes MOAP-1, VEGF, TRAF 6, CASP 2, E2F1 and CASP 5 in Jurkat cells and FTY720 treated Jurkat cells. (**A**) Representative gel electrophoresis of PCR products. (**B**) Comparison of the mRNA levels of six selected genes detected by semi-quantitative RT-PCR to those from microarray data. The quantitation of the mRNA expression of the six genes is shown as the average ratio of band intensity to that of the internal control β–actin. Data are expressed as mean ± S.D. from three independent experiments.

**Figure 5:**
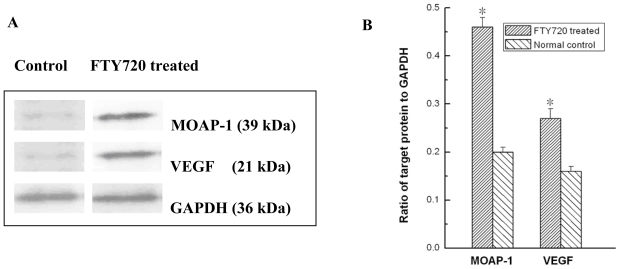
Western blotting analyses of MOAP-1 and VEGF protein expression in Jurkat cells and FTY720 treated Jurkat cells. (**A**) Representative Western blot. (**B**) Quantitation of the protein levels of MOAP-1 and VEGF in cells was shown as the average ratio of band intensity to that of the internal control GAPDH. Data are expressed as mean ± S.D. from three independent experiments. * *P* < 0.05 *versus* normal control cells.

To validate the microarray results, six genes including MOAP-1, VEGF, TRAF 6, CASP 2, E2F 1 and CASP 5 were chosen from the top of Table 2 as representatives of pro-apoptosis genes for RT-PCR analysis. The results showed that the mRNAs of all six selected genes were significantly up-regulated in FTY720 treated cells (Figure 4A). The levels of the selected gene expression (fold increase) were well matched between microarray and RT-PCR analyses (Figure 4B). To confirm the increase in MOAP-1 and VEGF expression at the protein level, Western blotting experiment was conducted (Figure 5A). Approximately 2.2 and 1.7 fold increase in MOAP-1 and VEGF protein expression was observed when Jurkat cells were treated with for 6 h (*P* < 0.05) (Figure 5B).

### 2.5. Discussion

The ability of FTY720 to initiate apoptosis in some leukemia cell lines or cancer cells has been reported. However, the apoptotic signaling mechanism in lymphocytes remains obscure. The present study examined the apoptotic signaling pathways with microarray analysis technique and drew a simplified diagram to show the potential signal transduction pathways mediated by FTY720.

Upregulation of expression of MOAP-1 and Bax by FTY720 has been observed in the present study, indicating that mitochondria related signaling pathways is likely to be involved in the FTY720-induced apoptotic process. MOAP-1 is a novel mitochondria enriched protein that associates with Bax upon stimulation. It facilitates translocation of Bax from cytosol to mitochondria which subsequently leads to the release of cytochrome *c* from the mitochondria [22]. It was found that up-regulation of MOAP-1 is not a consequence of apoptosis, but an early event in the apoptotic signaling process [23]. However, one study showed that death receptor signaling was related to Bax activation through binding to MOAP-1 [24], suggesting activation of MOAP-1 may be associated with cellular events other than apoptosis. Furthermore, enhanced expression of Bax and down regulated expression of bcl-2 after FTY720 treatment caused a marked decrease in the ratio of bcl-2 to Bax in Jurkat cells, suggesting further the importance of Bax in the FTY720-induced apoptotic process. It has been reported that FTY720 decreased the ratio of bcl-2/Bax in the human lymphocytes [17], which may support our observation above. Recently it was reported that FTY720-induced apoptosis strongly correlated with the inhibition of sphingosine kinase (SK) in LLC-PK1 cells, and subsequently reduced production of sphingosine-1-phosphate (S1P), a suppressor of Bax [25]. This may also contribute to FTY720-induced apoptosis in Jurkat cells.

Significantly upregulated expression of Smac (second mitochondria-derived activator of caspase) by FTY720 confirms that mitochondrial signaling pathways are key pathways involved in the FTY720-induced apoptotic process in Jurkat cells. Smac is a novel mitochondrial protein, which is released into the cytosol when a cell undergo apoptosis. It can promote apoptosis by binding to inhibitor of apoptosis proteins (IAPs) and subsequently activating caspases [26]. Over expression of Smac has been found in some tumor cells including gastric cancer cells, lung cancer cells, ovarian cancer cells [27], our data are in line with those previous studies. Another element of mitochondrial signaling pathways Apaf-1 (apoptotic protease activating factor 1) was also overexpressed by Jurkat cells in response to FTY720. Since Apaf-1 has been found to participate in the pathway of mitochondria-mediated apoptosis [28], it may be involved in FTY720 induced apoptosis in Jurkat cells.

Besides the genes in mitochondria related signaling pathways, some genes of the death receptor mediated apoptotic signal pathway such as tumor necrosis factor receptor superfamily member 10B (TNFRSF10B) and lymphotoxin alpha (LTA), and endoplasm mediated apoptotic signal pathway such as calcium channel voltage-dependent beta 2 subunit (CACNB 2) and calcium channel voltage-dependent beta 3 subunit (CACNB 3) were observed to be upregulated after FTY720 treatment, indicating that mitochondria apoptotic signal pathway is unlikely to be the only pathway involved in the FTY720-induced apoptosis. Similarly, genes of VEGF, tumor necrosis factor receptor-associated factors (TRAF 6), transcription factor E2F 1 and caspase 5 were also found to be significantly up-regulated in response to FTY720. However, it is rather difficult to draw a clear conclusion for their involvement in FTY720-induced apoptotic signaling process in Jurkat cells.

## 3. Experimental Section

### 3.1. Materials

Jurkat cells were obtained from the ATCC Corporation (Manassas, VA, USA). EZ-10 column DNA mini-preps kit was purchased from Bio Basic Inc. (East Markham, Ontario, Canada). Annexin V-FITC Apoptosis Detection Kit I was from BD Biosciences (San Diego, CA, USA). BCA Protein Assay Kit was from Pierce Endogen (Rockford, IL, USA). Rabbit anti-human modulator of apoptosis-1 (MOAP-1) polyclonal antibody was purchased from ProSci Incorporated (Poway, CA, USA), rabbit anti-human vascular endothelial growth factor (VEGF) polyclonal antibody was from Santa Cruz Biotechnology (Santa Cruz, CA, USA), and swine anti-rabbit IgG-HRP was from DakoCytomation (Produktionsvej, Glostrup, Denmark). Enhanced chemiluminescence reagent and Cy3 or Cy5-dUTP was purchased from Amersham Pharmcia Biotechnology (Piscataway, NJ, USA). Prestained protein marker was from New England Biolabs (Beverly, MA, USA). 3-(4,5-dimethylthia-zol-2-yl)-2,5-diphenyl tetrazolium bromide (MTT) was from Bio Basic Inc. (Markham, Ontario, Canada). RPMI 1640 medium, fetal bovine serum and TRIzol reagent were purchased from Invitrogen Life Technologies (Carlsbad, CA, USA). FTY720 was from Cayman Chemical (Ann Arbor, MI, USA). Dimethyl sulfoxide and albumin bovine were from Amresco Inc. (Solon, Ohio, USA). Hoechst 33258 was from Sigma (St. Louis, MO, USA). RNeasy Mini Kit and QIAquick Nucleotide Removal kit were purchased from Qiagen (Hilden, Germany). TaKaRa High Fidelity RNA PCR Kit was purchased from TaKaRa Biotechnology (Dalian, China) and Taq PCR MasterMix was from Tiangen Biotech Co. (Beijing, China). The Human Apoptosis Microarray V 1.1 was from National Engineering Center for Biochip (Shanghai, China).

### 3.2. Cell Culture and FTY720 Treatment

Jurkat cells were grown in RPMI 1640 medium supplemented with 10% fetal bovine serum, penicillin (100 U/mL), streptomycin (100 U/mL), and glutamine (2 mM) in 25-cm^2^ tissue culture flasks at 37 °C in a 5% (v/v) CO_2_, water-saturated atmosphere. The cell viability was greater than 95% assessed by trypan blue staining. The medium was replaced every 2 days, and the cells were in logarithm phase for the experiments. FTY720 was dissolved in RPMI 1640 medium at concentrations ranging from 2 to 10 μM.

### 3.3. Viability Assay by MTT

The cells were resuspended in complete RPMI 1640 medium at a concentration of 5 × 10^4^ cell/mL. Cells (1 × 10^4^) were incubated in a 96-well flat-bottomed culture plate in 200 μL volumes with or without various concentrations (2 and 10 μM) of FTY720 for 12, 18 and 24 h, respectively, at 37 °C. Cell viability was assessed by MTT assay. Briefly, 20 μL of 5 mg/mL MTT was added to each well before the end of the FTY720 treatment, and incubated for a further 4 h. After incubation, the plates were centrifuged at 450 g for 5 min and the supernatants were discarded. Dimethyl sulfoxide (150 μL) was then added to each well for 10 min in the dark, followed by addition of formazan crystal solution. The absorbance was measured at 570 nm using an ELISA plate reader (Bio-Rad 680, USA).

### 3.4. Apoptosis AssaysHoechst 33258 staining

Cells (5 × 10^5^) were harvested after 2, 4 and 6 h incubation period and centrifuged at 400 g for 5 min. After washing with cold phosphate-buffered saline (PBS), cells were fixed in 50 μL of 3% polyformaldehyde solution for 10 min at room temperature, and then exposed to 15 μL of Hoechst 33258 in a final concentration of 16 μg/mL for 15 min in the dark. After washing, 10 μL of cell pellet was dropped on a glass slide and the image was observed under a fluorescence microscope (Nikon TE2000, Japan). The apoptosis in Jurkat cells was determined by the alteration of nuclear morphology and fluorescent density observed after staining with Hoechst 33258.

#### Flow cytometry analysis

Cells (1 × 10^6^) were harvested after 3 and 6 h incubation period and centrifuged at 450 g for 5 min at 4 °C. After washing twice with PBS, the cell pellet was suspended in 100 μL Annexin V binding buffer. Cells were incubated with 5 μL Annexin V-FITC and 5 μL propidium iodide (PI) for 15 min at room temperature away from light before addition of 400 μL Annexin V binding buffer. The cell pellet was analyzed using a FACScan flow cytometer (BD Biosciences, San Jose, CA). The early apoptotic cells (Annexin-FITC positive and PI negative) were located in the lower right quadrant. The late apoptotic or necrotic cells (Annexin-FITC positive and PI positive) were located in the upper right quadrant. Healthy cells (negative for both probes) were located in the lower left quadrant. The results are expressed as percentage of positively stained cells in total cells.

#### DNA fragmentation

Cells (2 × 10^6^) were harvested after 0, 1, 2, 4 and 6 h and centrifuged at 450 g for 5 min at 4 °C. The DNA extraction and purification was performed using the EZ-10 column mini-preps kit according to the manufacture’s instruction. Briefly, cells were suspended in 800 μL TBP Buffer and centrifugated at 800 g for 3 min. The cells were then mixed with 500 μL TBM Buffer and 3 μL proteinase K at 55 °C for 30 min. After centrifugation at 1200 g for 2 min, a mixture of the cell supernatant with 260 μL absolute ethanol was applied to the EZ-10 column. This was followed by addition of 30 μL elution buffer (2.0 mM Tris-Hcl, PH 8.0) to the column and incubated at 50 °C for 2 min. The column was spun at 2000 g for 1 min to elute DNA from the column. The DNA quantity was measured by UV absorption at A260 with a spectrophotometer (Beckman Coulter DU800, USA). DNA samples were electrophoresed on 2% agarose gels with SYBR Green I staining, and the bands were visualized by a Bio-Rad Gel Imaging System (Bio-Rad ChemiDoc XRS, USA).

### 3.5. Microarray Analysis

Cells (1 × 10^7^) were harvested 6 h after treatment with 10 μM FTY720, and centrifuged at 450 g for 5 min at 4 °C. Total RNA was prepared using TRIzol reagent, further purified with an RNeasy Mini Kit. Approximately 2 μg of total RNA was reverse-transcribed, amplified and labeled with either Cy3 (treated group) or Cy5 (untreated control). Four micrograms of labeled cDNA was hybridized to Human Apoptosis Microarray V 1.1, which consists of 458 genes involved in apoptosis, including death receptors and their ligands, caspases, Bcl-2 gene family, calpains, death kinases, granzymes, DNA fragmentation proteins, and some other genes, according to the manufacture’s instruction. After washing, the arrays were scanned using the Agilent GeneArrayTM scanner system (Hewlett-Packard, USA).

Three independent experiments were performed, and expression signals were converted into numerical data using the ImaGene software 5.5 (BioDiscovery Inc., CA, USA) and the raw data was saved as a Microsoft Excel file. Data were subsequently exported to Genespring version 7.0 (Silicon Genetics, Redwood City, CA, USA) for background subtraction based on negative controls and per spot and per chip intensity dependent normalization (non-linear or LOWESS normalization). These corrected, normalized signals can then be used to estimate the relative abundance of genes with the ratio of Cy-3 and Cy-5 signal intensities. Changes in gene expression were considered significant if the detection *P* value was less than 0.05 in at least two of three arrays, and the fold change was at least 2.0.

### 3.6. Semi-Quantitative RT-PCR Analysis

Semi-quantitative RT-PCR analysis was used to confirm expression of the six selected pro-apoptosis genes including MOAP-1, VEGF, TNF receptor-associated factor 6 (TRAF 6), caspase 2 (CASP 2), E2F transcription factor 1 (E2F 1) and caspase 5 (CASP 5), which were up-regulated more than 5-fold following FTY720 treatment in Jurkat cells in the microarray analysis. Jurkat cells (6 × 10^6^) were harvested at 6 h after treatment with 10 μM FTY720 and total RNA was prepared with TRIzol reagent. The cDNA synthesis was performed with TaKaRa High Fidelity RNA PCR kit according to the manufacturer’s instruction: 5 μL of 2 × *Bca* 1st buffer, 2 μL of MgSO_4_ (25 mM), 0.5 μL of dNTP mixture (10 mM), 0.25 μL of RNase inhibitor (40 U/μL), 0.5 μL of *Bca* PLUS RTase (42 U/μL), 0.5 μL of Random 9-mers and 1.2 μL of total RNA (400 ng) were mixed. cDNA synthesis was performed in 1 cycle: 65 °C for 1 min, 30 °C for 1 min, gradually raise to 60–65 °C for 15 min, 98 °C for 5 min and 5 °C for 5 min. The amount of cDNA product was measured by a UV spectrophotometer (Beckman coulter DU800, USA). The PCR reaction was performed with Taq PCR MasterMix according to the manufacturer’s instruction: 2 μL of cDNA (1 μM), 1 μL of sense primer (10 μM), 1 μL of antisense primer (10 μM), 12.5 μL of 2 × Master Mix and 8.5 μL of ddH_2_O was added to the PCR tubes. The primer sequences were designed with Primer Premier 5.0 software. All primers were synthesized by Invitrogen (Shanghai, China). The primer pairs and the predicted sizes of the amplified PCR products and the PCR annealing temperatures are shown in Table 1. The PCR conditions included one initial denaturation at 95 °C for 15 min, followed by 35 cycles of the following conditions: 94 °C for 1 min, 50–65 °C for 30 s, 72 °C for 30 s and a final extension at 72 °C for 10 min. All PCR reactions were performed using a Bio-Rad Gradient Cycler (Bio-Rad PTC-200, USA). Eight microliters of PCR product was electrophoresed on 1% agarose gels, stained with SYBR Green I, and visualized by a Bio-Rad Gel Imaging System (Bio-Rad ChemiDoc XRS, USA). The gels were analyzed by using Quantity One 1-D Analysis Software (Bio-Rad, USA). The relative quantities of gene expression were presented as the ratio between the intensity of MOAP-1, VEGF, TRAF 6, CASP 2, E2F 1, CASP 5 band and that of the housekeeping gene β-actin.

### 3.7. Western Blotting Analysis

Protein levels of MOAP-1 and VEGF in Jurkat cells treated with or without FTY720 were examined by Western blot. Cells (6 × 10^6^) were collected at 6 h after treatment with 10 μM FTY720 and centrifuged at 450 g for 5 min at 4 °C. After washing with cold PBS, the cell pellet was lysed with extraction buffer (50 mM Tris-HCl, 150 mM NaCl, 1% NP-40, 0.25% NaDC, 1 mM EDTA, 1 mM PMSF, 1 mM Na_3_VO_4_ and 10 μg/mL aprotinin) for 30 min at 4 °C. The cell lysate was then centrifuged at 2,200 g for 30 min, and the supernatant was collected. Protein concentration was determined by using a BCA protein assay kit. Thirty micrograms of denatured protein of each sample was separated by 12% sodium dodecyl sulfatepolyacrylamide gel electrophoresis (SDS-PAGE), transferred electrophoretically to polyvinylidene fluoride (PVDF) membranes, and blocked with 5% albumin bovine overnight. The membranes were incubated with rabbit anti-human MOAP-1 polyclonal antibody (1:500 in TBST, TBST: 12.5 mM Tris/HCl, pH 7.6, 137 mM NaCl, 0.1% Tween 20) or rabbit anti-human VEGF polyclonal antibody (1:1000 in TBST) for 2 h at room temperature. After washing with TBST for 3 times, the membranes were incubated with swine anti-rabbit IgG-HRP (1:2000) for 1 h at room temperature. The protein bands on the membranes were visualized by using enhanced chemiluminescence reagent according to the manufacturer’s instruction. A prestained protein marker was used as molecular weight marker. Equivalent protein loading and transfer efficiency were verified by staining for glyceraldehyde-3-phosphate dehydrogenase (GAPDH). The bands were analyzed with a Bio-Rad Gel Imaging System (Bio-Rad ChemiDoc XRS, USA) and Quantity One 1-D Analysis Software (Bio-Rad, USA).

### 3.8. Statistical Analysis

Data were expressed as mean ± S.D. for the number of experiments indicated. All statistics were performed using SPSS 12.0 software. Statistically significant differences between groups were determined using a multivariate analysis of variance with post hoc test. Comparisons between two groups were performed by Student’s *t* test for parametric data and Mann-Whitney U test for nonparametric data. Correlation was calculated using Spearman’s non-parametric test. *P* < 0.05 was considered statistically significant.

## 4. Conclusions

**Figure 6:**
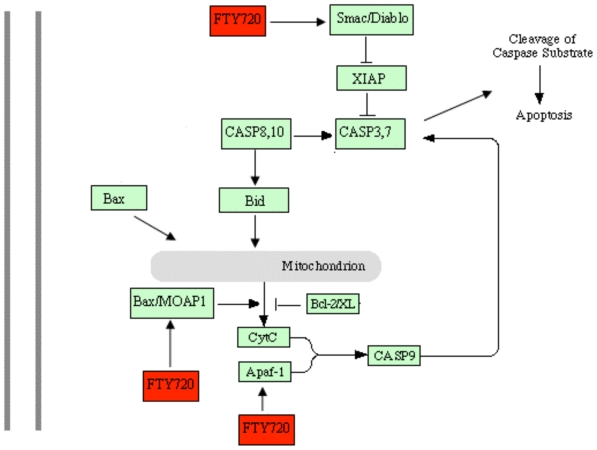
Simple diagram of FTY720 action in the mitochondria mediated apoptotic pathway. FTY720 up-regulated the expression of the genes MOAP-1, Bax, Smac and Apaf-1, and down-regulated Bcl-2 expression. →shows activation. ⟙ shows inhibition.

In conclusion, FTY720 is capable of inducing Jurkat cell apoptosis. The signaling mechanism of FTY720-induced apoptosis is most likely through activation of mitochondria signaling pathways. Based on previous findings and our current results, we manage to draw a simplified diagram showing the potential signal transduction mechanisms of FTY720-induced apoptosis (Figure 6).

## Figures and Tables

**Table 1:** Primer sequences and annealing temperature of the genes in RT-PCR.

Gene	Sense primer (5′–3′)	Antisense primer (5′–3′)	Product size (bp)	Annealing temperature (°C)
MOAP-1	GAGTCTCAGGGAACCACGAAG	GGCACAGAAACGACAAAGGGC	155	60
VEGF	CCCACTGAGGAGTCCAACA	CAAATGCTTTCTCCGCTCT	174	57
TRAF6	CCTGTTGTGATTCATAGCC	GCATCCATTATCTCTTCGTG	242	55
CASP2	TCCACCCCCACCACTCTTGACT	TGGCTTGCCTTCTTCCCTCTT	304	58
E2F1	TACCCCAACTCCCTCTACC	TCTGTCTCCCTCCCTCACT	121	62
CASP5	GTCTAAAGGACAAACCCAAGG	TGTGAAGAGATGAGTGCCAAG	109	57
β–actin	TCATGAAGTGTGACGTGGACATC	TCTAGTTCTAGTAACGAGGAGGAC	156	60

Six pro-apoptosis genes including MOAP-1, VEGF, TRAF 6, CASP 2, E2F 1 and CASP 5 were used to semi-quantitative RT-PCR analysis to confirm their expression in the microarray analysis, which were up-regulated more than 5-fold following FTY720 treatment in Jurkat cells in the microarray analysis. The primer pairs and the predicted sizes of the amplified PCR products and the annealing temperatures of PCR were listed. The primer sequences were designed with Primer Premier 5.0 software. β–actin is the housekeeping gene for the RT-PCR analysis.

**Table 2:** List of the apoptosis related genes that were significantly altered by FTY720 treatment in Jurkat cells. Total RNA from Jurkat cells treated with 10 μM FTY720 for 6 h was extracted for microarray analysis. Representative genes were significantly up-regulated (3-fold change) and down-regulated (2-fold change) by FTY720 in Jurkat cells (out of 64 significantly regulated genes by FTY720 in Jurkat cells in microarray analysis).

Gene symbol	Gene name	Fold change	P value
**Pro-apoptosis genes**			
MOAP1	Modulator of apoptosis 1	10.91	8.53E-10
VEGF	Vascular endothelial growth factor	7.72	4.01E-05
TRAF6	TNF receptor-associated factor 6	6.93	1.31E-05
CASP2	Caspase 2, apoptosis-related cysteine protease (neural precursor cell expressed, developmentally down regulated 2)	5.82	8.93E-10
E2F1	E2F transcription factor 1	5.68	1.25E-06
CASP5	Caspase 5, apoptosis-related cysteine protease	5.06	1.17E-05
Mdm4	Mdm4, transformed 3T3 cell double minute 4, p53 binding protein (mouse)	4.72	0.00011
BAX	BCL2-associated X protein	4.38	3.73E-05
BNIP3L	BCL2/adenovirus E1B 19kD interacting protein 3-like	4.18	0.00014
LTA	Lymphotoxin alpha (TNF superfamily, member 1)	3.64	8.12E-05
TUCAN	Tumor up-regulated CARD -containing antagonist of caspase 9	3.01	8.11E-07

**Anti-apoptosis genes**			
IGF2	Insulin-like growth factor 2 (somatomedin A)	6.17	1.81E-05
JUN (AP-1)	V-jun sarcoma virus 17 oncogene homolog (avian)	4.83	0.00017
BIRC1	Baculoviral IAP repeat-containing 1	4.07	6.0E-09
FKSG2	Apoptosis inhibitor	3.71	9.85E-08
BCL2	B-cell CLL/lymphoma 2	0.45	0.000377
RAD23B	RAD23 homolog B (S. cerevisiae)	0.48	0.000135
